# Desmopressin Stimulates Nitric Oxide Production in Human Lung Microvascular Endothelial Cells

**DOI:** 10.3390/biom12030389

**Published:** 2022-03-02

**Authors:** Bianca Maria Rotoli, Rossana Visigalli, Francesca Ferrari, Marianna Ranieri, Grazia Tamma, Valeria Dall’Asta, Amelia Barilli

**Affiliations:** 1 Laboratory of General Pathology, Department of Medicine and Surgery, University of Parma, 43125 Parma, Italy; biancamaria.rotoli@unipr.it (B.M.R.); rossana.visigalli@unipr.it (R.V.); francesca.ferrari@unipr.it (F.F.); valeria.dallasta@unipr.it (V.D.); 2 Department of Bioscience, Biotechnology and Biopharmaceutics, University of Bari, 70125 Bari, Italy; marianna.ranieri@uniba.it (M.R.); grazia.tamma@uniba.it (G.T.)

**Keywords:** desmopressin, human endothelium, nitric oxide, NOS2

## Abstract

Desmopressin (dDAVP) is the best characterized analogue of vasopressin, the endocrine regulator of water balance endowed with potent vasoconstrictive effects. Despite the use of dDAVP in clinical practice, ranging from the treatment of nephrogenic diabetes insipidus to bleeding disorders, much remains to be understood about the impact of the drug on endothelial phenotype. The aim of this study was, thus, to evaluate the effects of desmopressin on the viability and function of human pulmonary microvascular endothelial cells (HLMVECs). The results obtained demonstrate that the vasopressor had no cytotoxic effect on the endothelium; similarly, no sign of endothelial activation was induced by dDAVP, indicated by the lack of effect on the expression of inflammatory cytokines and adhesion molecules. Conversely, the drug significantly stimulated the production of nitric oxide (NO) and the expression of the inducible isoform of nitric oxide synthase, NOS2/iNOS. Since the intracellular level of cAMP also increased, we can hypothesize that NO release is consequent to the activation of the vasopressin receptor 2 (V2R)/guanylate cyclase (Gs)/cAMP axis. Given the multifaceted role of NOS2-deriving NO for many physio-pathological conditions, the meanings of these findings in HLMVECs appears intriguing and deserves to be further addressed.

## 1. Introduction

Desmopressin (1-deamino-8-D-arginin vasopressin; dDAVP) is a synthetic analogue of vasopressin (AVP). The latter, also called an “antidiuretic hormone” (ADH), is a natural hormone with potent vasoconstrictive effects and the key endocrine regulator of water balance [1].

In humans, the activity of AVP and its analogues depends on the binding to specific receptors on the cell surface, such as AVPR1A/V1aR, AVPR2/V2R, and AVPR1B/V1bR. They all are seven-transmembrane G-protein-coupled receptors. V1aR is mainly expressed on smooth muscle cells where its activation increases intracellular Ca^2+^ content via phosphatidyl-inositol intracellular cascade, leading to vasoconstriction. The stimulation of V2R in the kidney collecting duct, by activating adenyl cyclase, leads to the increased expression and synthesis of the aquaporin water channel AQP-2, causing water reabsorption from the urine. The expression of V1bR is limited to the anterior pituitary gland and the pancreas, where interaction with vasopressin induces corticotropic axis stimulation and insulin secretion [2].

Vasopressin and its analogues, due to their roles in osmoregulation, cardiovascular control, and homeostasis, have been used traditionally to treat pathological conditions, such as central diabetes insipidus and bleeding disorders; more recently, experimental evidence has suggested new scenarios of therapeutic applications, including cardiopulmonary resuscitation, septic shock, intraoperative hypotension, and portal venous hypertension [3]. The mechanism of action for vasopressin and its related compounds has been demonstrated in animal models, usually reflecting the activity in a clinical setting; however, in some cases, important differences exist among species, in particular for V2R-mediated responses [4].

Among the analogues of vasopressin that preferentially target V2R, desmopressin is the best-characterized [5]. dDAVP was initially developed for the treatment of nephrogenic diabetes insipidus (NDI); then, due to its ability to increase plasma levels of FVIII [6] and the von Willebrand factor (vWF), its use was extended to patients with bleeding disorders, such as mild and moderate hemophilia A, selected types of von Willebrand Disease, several forms of platelet dysfunction, and uremic bleeding [7]. More recently, the possible repositioning of the drug as an adjuvant agent in the treatment of cancer has been also proposed, due to its antiangiogenic and antimetastatic properties [8].

Despite the centrality of dDAVP in clinical practice, much remains thus far to be understood about the impact of the drug on the integrity of endothelium [9]. Recently, Lopez and colleagues showed that the AVP–V2R axis is critical in the pathophysiology of severe microvascular hyperpermeability in sepsis, raising particular attention toward the dynamics of V2R activation in the microvasculature that may directly impact the outcome of septic patients [10]. In this context, we evaluate the effects of treatment with V2R agonist desmopressin on the viability and function of human pulmonary microvascular endothelial cells.

## 2. Materials and Methods

### 2.1. Cell Cultures and Experimental Treatment

The study has been performed employing Primary Human Lung Microvascular Endothelial Cells (HLMVEC) from Neo-Biotech (cat. # NB-11-0019; distributed by CliniSciences, Guidonia Montecelio, Italy), isolated from normal human peripheral lung tissue. According to the supplier’s instructions, the cells were grown on plasticware coated with Attachment Factor™ (Neo-Biotech) in Complete Serum Cell medium (cat. # NB-11-0046; Neo-Biotech) added with 10 mL of CultureBoost (Neo-Biotech), and maintained at 37 °C in a 95% humidified atmosphere with 5% CO_2_ in air. For the experiments, cells were seeded in multiwell plates and incubated with different concentrations of 1-deamino-8-D-arginine vasopressin (dDAVP) for different times, as indicated in the Figure legends.

### 2.2. Cell Viability Assay

Cells were seeded in complete growth medium in 96-well plates at a density of 5 × 10^3^ cells/well. After the treatment for 1, 24 or 48 h with 1 and 100 nM dDAVP, cell viability was tested by measuring LDH release in the extracellular medium with a CytoTox96^®^ Non-Radioactive Cytotoxicity assay (Promega, Milano, Italy), according to the manufacturer’s instructions.

### 2.3. RT-qPCR Analysis

mRNA expression has been analyzed through RT-qPCR, as previously described [11], upon incubation of HLMVEC with 1 and 100 nM dDAVP for 1 or 16 h. 1 μg of cDNA was obtained through the reverse transcription of total RNA with the RevertAid First Strand cDNA Synthesis Kit (Thermo Fisher Scientific, Milano, Italy). qPCR was then performed on a StepOnePlus Real-Time PCR System (Thermo Fisher Scientific) by employing specific forward/reverse primer pairs (Table 1) and SYBR™ Green or TaqMan Gene Expression Master Mix (Thermo Fisher Scientific).

The expression level of genes coding for vasopressin receptors (AVPR1A and AVPR2) was determined using the formula 2ΔCt (where ΔCt = Ct_RPL15_ − Ct_gene of interest_) [12], after normalization for the housekeeping gene (Ribosomal like protein 15, RPL15, Gene ID: 6138). The amount of the different genes upon treatment with dDAVP was, instead, calculated with the ∆∆Ct method [11] and expressed, relatively to RPL15, as fold change of control untreated cells (=1).

### 2.4. Western Blot Analysis

For the determination of protein expression, cells either untreated or incubated for 30 min with 1 and 100 nM dDAVP were lysed in LDS sample buffer (Thermo Fisher Scientific) and Western Blot analysis was performed as described [13]. Briefly, 20 μg of proteins were separated on Bolt™ 4–12% Bis-Tris mini protein gel (Thermo Fisher Scientific) and electrophoretically transferred to PVDF membranes (Immobilione-P membrane, Merck). Membranes were incubated for 1 h at RT in TBST (50 mM Tris-HCl pH 7.5, 150 mM NaCl; 0.1% Tween) containing 5% non-fat dried milk, then incubated overnight at 4 °C in TBST added with 5% BSA and anti-AVPR1A (Thermo Fisher Scientific), anti-AVPR2 (MyBiosource, distributed by Aurogene S.r.l, Roma, Italy) anti-phospho-eNOS (Ser1177) (Cell Signaling Technology, distributed by Euroclone, Pero, Italy) purified rabbit polyclonal antibodies (1:2000). Actin, detected with a monoclonal antibody (1:2000; Merck, Milano, Italy), was employed as internal standard. Immunoreactivity was visualized with SuperSignal™ West Pico Plus Chemiluminescent HRP Substrate (Thermo Fisher Scientific). Western Blot images were captured with iBright FL1500 Imaging System (Thermo Fisher Scientific) and analyzed with iBright Analysis Software.

### 2.5. Determination of Nitric Oxide Production

The production of nitric oxide (NO) upon incubation with 1 and 100 nM dDAVP for 48 h was determined through a fluorimetric approach, based upon the production of the fluorescent molecule 1-(H)-naphtotriazole from 2,3-diaminonaphthalene (DAN) in acid environment, as previously described [14]. Briefly, 100 μL of cell medium were transferred in wells of a black 96-well plate with a clear bottom (Corning, distributed by Euroclone, Pero, Italy) and added with 20 μL DAN (0.025 mg/mL in 0.31 M HCl). After 10 min at room temperature, the reaction was stopped with 20 μL of 0.7 M NaOH and fluorescence was read with the EnSpire^®^ Multimode Plate Reader (PerkinElmer, Milano, Italy). Standard sample were run in parallel, by diluting a solution of 1 mM sodium nitrite in the same medium; blank, corresponding to the value measured in a cell-free sample, was subtracted from all the measurements. Nitrite production is expressed in nmoles per ml of extracellular medium (μM). When required, 100 μM N^6^-(1-iminoethyl)-L-lysine (NIL) was employed as NOS2 inhibitor [15].

### 2.6. Arginine Uptake

Arginine transport across the plasma membrane of dDAVP-treated cells was measured as already described [16] in cells incubated for 8 h with 100 nM dDAVP. After two washes in pre-warmed transport buffer (Earle’s Balanced Salt Solution (EBSS) containing (in mM) 117 NaCl, 1.8 CaCl_2_, 5.3 KCl, 0.9 NaH_2_PO_4_, 0.8 MgSO_4_, 5.5 glucose, 26 Tris/HCl, adjusted to pH 7.4), cells were incubated in the same solution additioned with L-[^3^H]arginine (50 μM, 5 μCi/mL). After 30 s, transport buffer was removed, and the experiment terminated by two rapid washes (<10 s) in ice-cold urea (300 mM). The ethanol soluble pool was extracted from monolayers and radioactivity in cell extracts was determined by using a MicroBeta2^®^ liquid scintillation spectrometer (PerkinElmer). Arginine uptake was normalized for protein content, determined directly in each well by using a modified Lowry procedure [17], and expressed as nmol/mg of protein/min.

### 2.7. Determination of Arginine Intracellular Concentration

The measurement of intracellular arginine was performed as already described [18]. Cells, grown on 24-well trays, were incubated with 1 and 100 nM dDAVP for 24 h; at the end cells were rapidly washed with ice-cold PBS and the intracellular pool was extracted with a 10 min-incubation in 200 μL of ethanol at 4 °C. After freeze-drying, samples were suspended in 150 μL of Lithium Loading Buffer (Biochrom, distributed by Erreci, Opera, Italy) and the intracellular content of each amino acid specie was determined by means of HPLC analysis with a Biochrom 30 amino acid analyzer (Biochrom) employing a high resolution lithium column and lithium buffers for elution (Biochrom). The column effluent was mixed with EZ Nin Reagent kit (Biochrom), passed through the high-temperature reaction coil, and read by the photometer unit at both 570 nm and 440 nm. Arginine was identified by running standard samples (Biochrom) in parallel. For the determination of arginine intracellular concentration, cell volume was estimated from the distribution of [^14^C]urea, as already described [19]; calculated cell volumes corresponded to 10.09 ± 0.18 μL/mg of protein and 8.89 ± 0.6 μL/mg of protein for control untreated cells and cells incubated for 24 h with dDAVP, respectively.

### 2.8. Fluorescence Resonance Energy Transfer Measurements

To evaluate intracellular cAMP changes, fluorescence resonance energy transfer (FRET) technology was applied. For FRET experiments, cells, grown on coverslips, were incubated for different times with 100 nM dDAVP, then transfected with a plasmid encoding the H96 sensor containing the cAMP-binding consensus motif of EPAC1 embedded between the cyan fluorescent protein (CFP) and cp173Venus-Venus as previously shown [20]. ECFP- and/or EYFP-expressing cells and detection of FRET was performed using an inverted TE2000-S microscope (Nikon Eclipse microscope, Tokyo, Japan). Each image was corrected and analyzed as previously shown [21].

### 2.9. Intracellular Calcium Measurements

Intracellular calcium measurements were performed as previously shown [22] in cells incubated for different times with 100 nM dDAVP. Briefly, cells grown on coverslip were loaded with 4 μM Fura-2AM for 15 min at 37 °C in DMEM/F-12. Coverslips with dye-loaded cells were mounted in a perfusion chamber (FCS2 Closed Chamber System, BIOPTECHS, Butler, PA, USA) and maintained in Ringer’s solution (containing in mM: 140 NaCl, 5 KCl, 1 MgCl_2_, 10 HEPES, 5 glucose, 1.8 CaCl_2_, pH 7.4). Fluorescence measurements were performed using an inverted TE2000-S microscope (Nikon Eclipse microscope). The ratio of intensities at 340 and 380 nm was plotted and calculated as change in fluorescence. In detail, stimulation with dDAVP was compared in the same cell type to those obtained after stimulation with a maximal dose of the calcium-mediated agonist ATP (100 μM) that was used as an internal control (100%). Intracellular calcium level was measured at steady-state and calibrated as described by Grynkiewicz [23]. Each sample was calibrated by the addition of 5 μM ionomycin in presence of 1 mM EGTA (Rmin) followed by 5 μM ionomycin in 5 mM CaCl_2_ (Rmax).

### 2.10. Statistical Analysis

The statistical analysis was performed using GraphPad Prism^®^ 9 (GraphPad Software version 9, GraphPad, San Diego, CA, USA). All data were analyzed with a two tailed Student’s *t*-test for unpaired data or with One-sample t test, as specified in the figure legends.

### 2.11. Materials

L-[2,3,4-3H]-monohydrochloride arginine (54.5 mCi/mmol) was obtained from PerkinElmer. Unless otherwise specified, all chemical and reagents were from Merck.

## 3. Results

**Figure 1:**
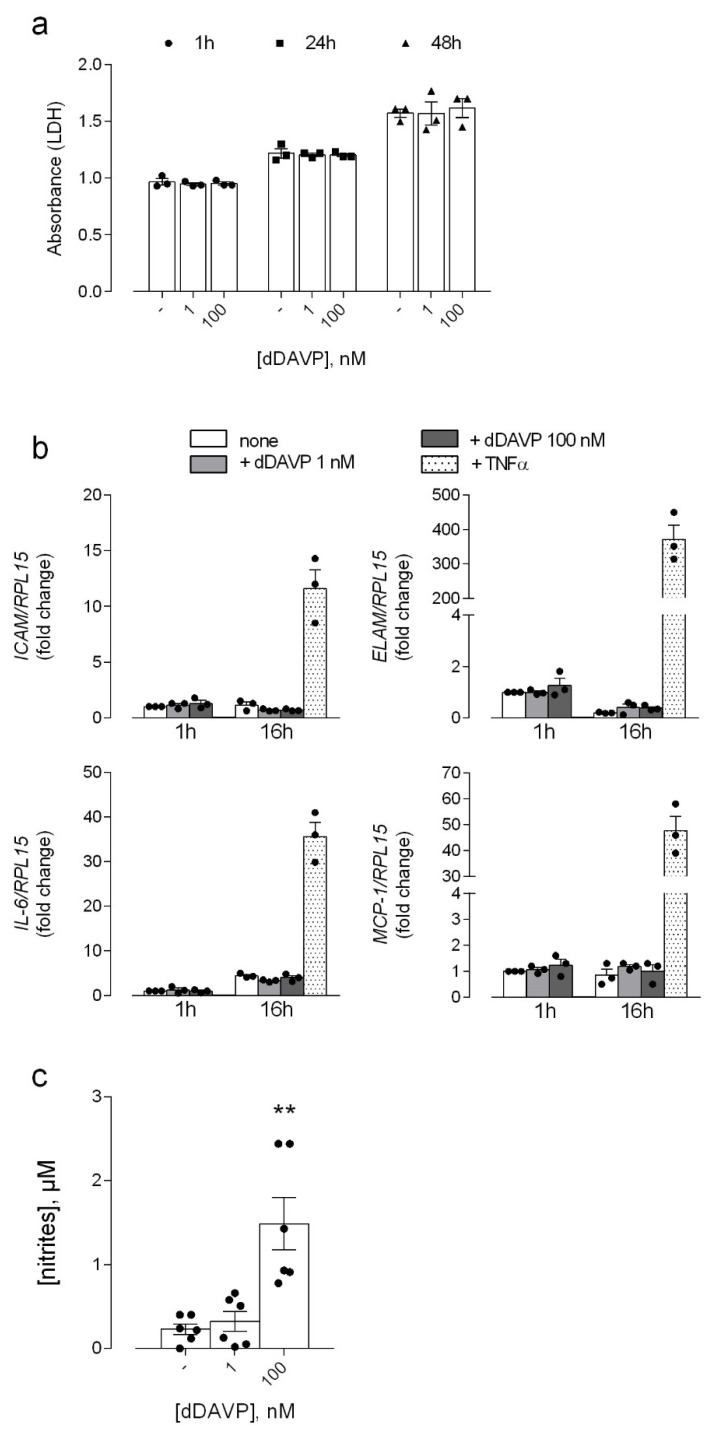
Effects of desmopressin on the viability and activation of microvascular endothelial cells. HLMVEC were incubated for the indicated times with different concentrations of dDAVP, as shown. (**a**). After the treatment, LDH release was assayed in the incubation medium as described in Methods. Data are the means ± SEM of four independent experiments each performed in triplicate. (**b**). The expression of the indicated genes was measured by means of RT-qPCR with the ∆∆Ct method and normalized for that of the housekeeping gene RPL15 (see Material and Methods). The stimulation with 10 ng/mL TNFα was employed for comparison, as positive control. (**c**). The production of NO was assessed through the quantitation of nitrites in the incubation medium, as described in Material and Methods. Data are means ± SEM of six independent determinations. ** *p* < 0.01 vs. control (-) with a two tailed Student’s *t*-test for unpaired data.

The aim of the present study has been to address the impact of the vasopressin analog desmopressin (dDAVP) on endothelial integrity, that is an issue, to date, only poorly addressed [9]. To this end, we evaluated the effects of short- (1 h) and long-term (24 and 48 h) incubations with the vasopressor on the viability and function of human lung microvascular endothelial cells (HLMVECs). Since peak plasma concentration of desmopressin in treated patients is approximately 4–10 pg/mL (3.7–9.3 pmol/L) [24], both low (1 nM) and high (100 nM) doses of dDAVP were employed in our experiments, so as to test the effects of physiological or slightly supra-physiological amounts of the drug. As for the cytotoxic effects of the drug, these were monitored by measuring the release of lactate dehydrogenase (LDH) in the extracellular medium. As shown in Figure 1a, despite a slight time-dependent increase of LDH in the incubation medium, no change was induced by dDAVP at any concentration at any time, thus excluding any cytotoxic effect of this compound, neither acute nor chronic.

The effects of the drug were, then, evaluated on the possible induction of an activated phenotype in the endothelium, by addressing the expression of adhesion molecules and inflammatory cytokines, as well as nitric oxide production; the stimulation with Tumor Necrosis Factor α (TNFα) was employed for comparison as positive control. The results obtained (Figure 1b) clearly demonstrate that the treatment with dDAVP does not modify the expression of any marker of endothelial activation, i.e., the adhesion molecules ICAM-1 and E-selectin (ELAM), the interleukin 6 (IL-6) and the chemokine monocyte chemoattractant protein-1 (CCL2/MCP-1). Instead, a significant accumulation of nitrites in the incubation medium was evident, with the extracellular concentration that raised from 0.23 ± 0.06 μM in control cells to 1.48 ± 0.31 μM after 48 h in the presence of 100 nM dDAVP (Figure 1c), thus indicating an increased production of nitric oxide (NO) under this condition.

**Figure 2:**
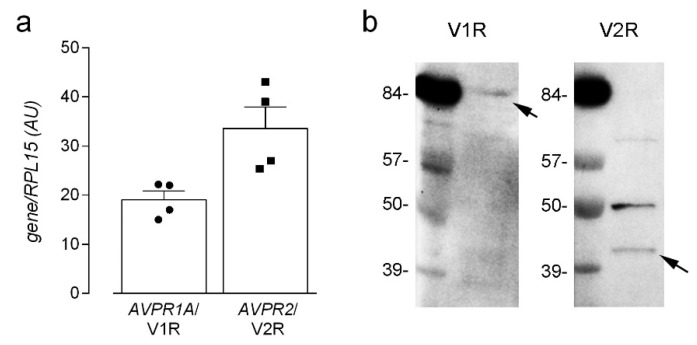
Expression of vasopressin receptors in microvascular endothelial cells. The expression of mRNA (**a**) and protein (**b**) for *AVPR1A*/V1R and *AVPR2*/V2R receptors was assessed in HLMVEC by means of RT-qPCR with the ∆∆Ct method and normalized for that of the housekeeping gene RPL15 (see Material and Methods). and Western Blot analysis, respectively, as described in Methods. Data in (**a**) are means ± SEM of four determinations, each performed in duplicate, while a representative blot is shown in (**b**) that, repeated three times, gave comparable results.

To explore the cellular and molecular mechanisms underlying this induction, we first checked the isoforms of vasopressin receptors actually expressed in our cell model, given the contradictory results shown in literature. Indeed, while the expression of V2R in human endothelial cells is well-established [25], the presence of V1Rs is still controversial [25,26]. Moreover, the selectivity of dDAVP for V2R has been established on the basis of results obtained in rats, but, in humans, affinity values are consistent with the interaction also with V1Rs [5]. In our hands, the mRNA for both receptors has been detected in HLMVECs (Figure 2a); consistently, bands corresponding to V1aR and V2R have been evidenced by means of Western Blot analysis, pointing to the presence of both proteins in lung microvascular endothelium (Figure 2b). As a result, the contribution of V1aR, beside that of V2R, to the observed induction of NO synthesis by desmopressin in our cell model cannot be excluded.

**Figure 3:**
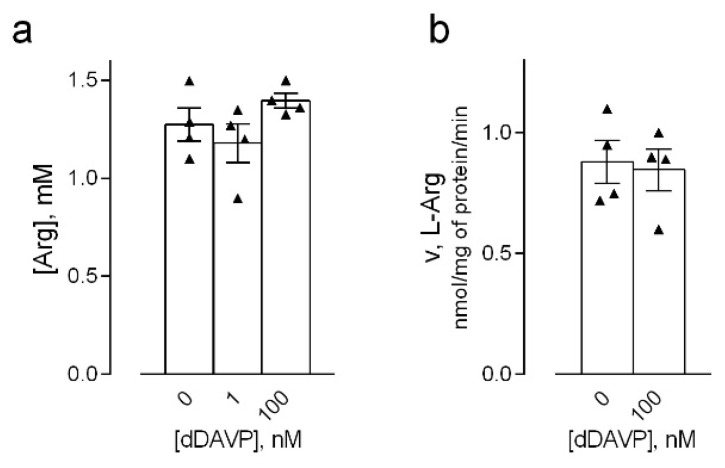
Arginine availability in desmopressin-treated human microvascular endothelial cells. HLMVEC were maintained for 24 h in the presence of the indicated concentrations of dDAVP. (**a**) Arginine intracellular concentration was measured through HPLC analysis after ninhydrin derivatization (see Material and Methods). Data are means ± SEM of three independent determinations. (**b**) Arginine uptake was assayed with a 30-s incubation of the cells in EBSS containing L-[^3^H]arginine (100 μM, 4 μCi/mL), as described in Material and Methods. Data are means ± SD of three determinations in a representative experiment that, repeated three times, gave comparable results.

Next, the effects of the drug in HLMVEC were addressed on both the expression of NOS2/iNOS and NOS3/eNOS, and on the intracellular availability of the NO obliged precursor arginine, so as to address more precisely the mechanisms responsible for the induction of NO production in our cell model. As far as the intracellular concentration of arginine is concerned, this did not differ between control and dDAVP-treated cells (Figure 3a). Accordingly, the treatment with 100 nM dDAVP for 24 h did not modify the transmembrane uptake of the amino acid (Figure 3b), excluding that the observed induction of nitric oxide production might be due to an increased availability of the precursor.

**Figure 4:**
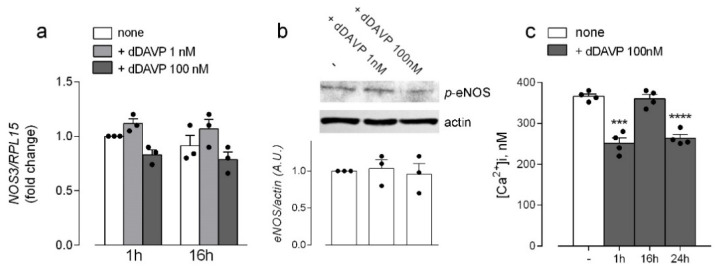
Effects of desmopressin on the expression and activity of endothelial nitric oxide synthases in microvascular endothelial cells. HLMVEC were maintained in the presence of the indicated concentrations of dDAVP. (**a**) The expression of NOS3 was measured after 1 and 16 h with RT-qPCR and normalized for that of the housekeeping gene RPL15. Data are means ± SEM of three experiments, each performed in duplicate. (**b**) The phosphorylation of eNOS in Ser1177 was monitored by means of Western Blot analysis, as described in Material and Methods. A representative blot is shown that, repeated three times, gave comparable results; the graph represents the mean ± SEM of the results from the densitometric analyses of the different experiments (**c**) Intracellular calcium levels were measured in cells treated for the indicated times at steady-state and calibrated as described in Material and Methods. Data are mean ± SEM of four independent determinations. *** *p* < 0.001, **** *p* < 0.0001 vs. control (-), with a two tailed Student’s *t*-test for unpaired data.

Similarly, no change was observed in the expression of the mRNA for the endothelial isoform NOS3/eNOS, nor in the phosphorylation of the enzyme at the activation site Ser1177 [27] (Figure 4a,b). Consistently, the intracellular content of calcium, which is among the activating factors of the enzyme [28], was significantly lowered, rather than increased, by both short- and long-term incubations with 100 nM dDAVP (Figure 4c).

**Figure 5:**
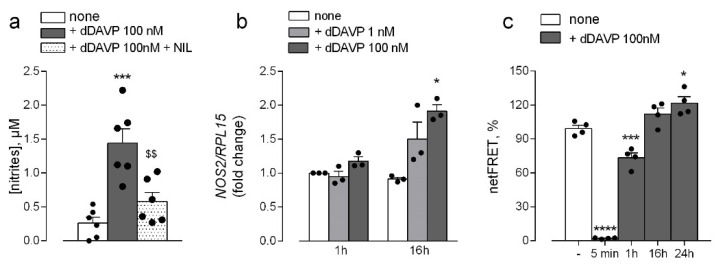
Effects of desmopressin on the expression and activity of inducible nitric oxide synthases in microvascular endothelial cells. HLMVEC were maintained in the presence of the indicated concentrations of dDAVP. (**a**) The production of NO was assessed through the quantitation of nitrites in the incubation medium upon treatment for 48 h in the presence of iNOS inhibitor N^6^-(1-iminoethyl)-L-lysine (NIL; 100 μM). Data are means ± SEM of six independent determinations. (**b**) The expression of NOS2 was measured after 1 and 16 h with RT-qPCR and normalized for that of the housekeeping gene RPL15. Data are means ± SEM of three experiments, each performed in duplicate. (**c**) After the treatment, cells were transiently transfected with the EPAC-based FRET sensor target to cytosol (H96) or specifically to mitochondria (4mtH30) and the FRET analysis was performed as described (see Methods). Bars represent the mean ± SEM of four independent experiments. * *p* < 0.01, *** *p* < 0.001, **** *p* < 0.0001 vs. none, ^$$^ *p* < 0.01 vs. dDAVP with a two tailed Student’s *t*-test for unpaired data.

Instead, the dDAVP-dependent induction of nitric oxide release was prevented by the addition to the incubation medium of iNOS inhibitor NIL (Figure 5a). Accordingly, a slight but significant increase was observed in the amount of NOS2/iNOS mRNA upon incubation for 16 h with the highest dose of the drug (Figure 5b). In line with this finding, also a significant increase of cyclic AMP (cAMP) release was rapidly (5 min) and transiently observed upon incubation of endothelial cells with 100 nM dDAVP, as demonstrated by the decrease of netFRET values (Figure 5c). Since cAMP is among the stimuli responsible for the regulation of NOS2 expression in many cell types [29], this finding further supports a role for the inducible isoform of the enzyme in the desmopressin-mediated stimulation of NO synthesis in HLMVEC.

## 4. Discussion

Despite the large use in the clinical practice, little is still known about the impact of the V2R-agonist desmopressin (dDAVP), a vasopressin analogue, on endothelial integrity [9]. In this field, the aim of the present study has been to address the effects of dDAVP on the viability and function of human lung microvascular endothelial cells (HLMVECs).

Literature evidences have recently indicated that short-term incubations (1, 3, 6 h) with dDAVP have no cytotoxic effect on human endothelium in vitro [10]. Consistently, we observed here no change in cell viability at any concentration tested at any time, even for longer incubations, excluding both acute and chronic cytotoxic effects. Similarly, no change was observed in the expression of any marker of endothelial activation. Instead, the incubation of HLMVECs for 48 h with 100 nM dDAVP resulted in an increased production of nitric oxide (NO).

A similar stimulation of NO release by desmopressin has been described in years both in vivo and in vitro, e.g., in human patients with heart failure [30], microperfused rabbit afferent arterioles [31], inner medullary collecting duct of the Brattleboro rat [32], and human umbilical vein endothelial cells (HUVEC) treated with 1 μM dDAVP after heterologous expression of V2R [33]. In those contributions, controversial conclusions were reached when addressing the isoform of nitric oxide synthase (NOS) specifically involved in NO production. Kaufmann and colleagues, indeed, referred the desmopressin-dependent stimulation of NO production by V2R-transfected HUVEC to the activation of endothelial NOS3/eNOS via the phosphorylation of Ser1177 [33]; conversely, a significant increase of NOS2/iNOS was detected by van Balkom et al. upon infusion of rat collecting ducts with dDAVP [32]. In our hands, no change in the expression of the mRNA for NOS3, nor in the phosphorylation of the enzyme was observed, excluding a role for eNOS in the observed increase of NO; consistently, intracellular calcium levels even decreased when cells were incubated in the presence of desmopressin. However, a transient recovery of calcium levels was observed after 16 h of incubation with the vasopressor. Intracellular calcium dynamics is, indeed, quite complex and intracellular calcium waves with peculiar features and frequencies at cellular and organ levels have been described in several systems upon interaction with hormones, including vasopressin [34,35]; among the others, several proteins, including plasma membrane receptors such as V1aR are known to be involved in the modulation of calcium homeostasis [36]. Interestingly, although the presence of V1Rs in human endothelial cells is quite controversial [25,26], our data demonstrate the expression of V1aR, besides V2R, in HLMVECs. As a result, we might speculate that, in human lung microvascular endothelial cells, the simultaneous activation of V1aR and V2R by desmopressin causes an early increase off calcium, which occurs within seconds, followed by a significant reduction of intracellular calcium level, possible making more sensitive to further intracellular calcium signals. Once verified, this hypothesis could give reason of the discrepancies between the findings by Kaufmann [37] and ours, which maybe referable to the different experimental timing adopted by the two groups to address the activation of NOS3/eNOS; the issue, hence, deserves to be further addressed.

Although we cannot exclude a contribution of the endothelial synthase in dDAVP-dependent stimulation of NO production, a more likely involvement of the inducible isoform is expected, based on the results obtained. Indeed, the simultaneous incubation with desmopressin and an inhibitor of iNOS prevented the increase of nitric oxide release, and, consistently, a slight but significant induction of NOS2/iNOS mRNA was observed upon treatment with the vasopressor, along with an evident increase of cyclic AMP (cAMP). Since cAMP acts as a known marker of V2R activation [38], as well as a modulator of NOS2 transcription [12], we can conclude that the induction of NO production and release driven by desmopressin in HLMVECs is mediated by the activation of NOS2/iNOS by dDAVP via the axis vasopressin receptor 2 (V2R)/guanylate cyclase (Gs)/cAMP.

The meaning of this induction in the microvasculature remains to be elucidated. The finding that iNOS-dependent NO exerts antimicrobial functions in vitro and in vivo has given NOS2 a role as master regulator of inflammatory response for long time [39]. However, despite the many contributions supporting a detrimental role for iNOS-derived NO, an increasing body of evidence now ascribe to NOS2 protective effects in the management of several pathological conditions [40]. The most likely hypothesis is, hence, that whether the induction of iNOS is beneficial or detrimental depends on many variables, such as the type of insult, the level and duration of expression, and the redox state of the tissue [41]. Interestingly, the activation of V2R in septic shock has been proposed to associate with adverse effects, such as selective vasodilation, prothrombosis, antidiuresis, and central nervous system changes [42]; as a consequence, a beneficial role has been recently ascribed to the inhibition of this receptor in the treatment of sepsis [10]. On these bases, the desmopressin-dependent induction of iNOS that we observed in HLMVECs appears particularly intriguing and its potential involvement in the adverse effects of V2R activation deserves to be addressed.

## Figures and Tables

**Table 1:** Sequences of the primer pairs employed for RT-qPCR analysis.

Gene/Protein Name (Gene ID)	Forward Primer	Reverse Primer
*AVPR1A*/V1aR (ID: 552)	CAGCGTGAAGTCCATTTCCC	GCAGACGATGTAAGCCGTCA
*AVPR2*/V2R (ID: 554)	CTAGTGGCTTGGGCCTTCTC	TAGGTGCCACGAACACCATC
*ICAM1*/ICAM1 (ID: 3383)	TGAACCCCACAGTCACCTATG	CTCGTCCTCTGCGGTCAC
*SELE*/ELAM (ID: 6401	ACCTCCACGGAAGCTATGACT	CAGACCCACACATTGTTGACTT
*IL6*/IL-6 (ID: 3569)	AACCTGAACCTTCCAAAGATGG	TCTGGCTTGTTCCTCACTACT
*CCL2*/MCP1 (ID: 6347)	CAGCCAGATGCAATCAATGCC	TGGAATCCTGAACCCACTTCT
*NOS2*/iNOS (ID: 4843)	Hs01075529_m1 (TaqMan^®^ Assay, ThermoFisher Scientific)	
*NOS3*/eNOS (ID: 4846)	TGGTACATGAGCACTGAGATCG	CCACGTTGATTTCCACTGCTG
*RPL15*/RPL15 (ID: 6138)	GCAGCCATCAGGTAAGCCAAG	AGCGGACCCTCAGAAGAAAGC

## Data Availability

The data presented in this study are contained within the article.
